# Anthony Fauci: a scientific adviser’s role from HIV to COVID-19

**DOI:** 10.2471/BLT.23.030123

**Published:** 2023-01-01

## Abstract

Anthony Fauci talks to Gary Humphreys about his achievements at the National Institute of Allergy and Infectious Diseases (NIAID), his experiences advising seven consecutive United States presidents, and the challenges faced in communicating scientific evidence.

*Q: You stepped down as director of the National Institute of Allergy and Infectious Diseases (NIAID) in December. What prompted that decision?*

A: There were different considerations but one of them was the fact that I was about to turn 82 in December and wanted to have a few years to pursue some interests outside the federal government while I still had the energy and passion required.

*Q: Was it a difficult decision to take?*

A: It was because I love the NIH (National Institutes of Health, of which NIAID is a division) and spent most of my career there having joined in 1968 after I completed my medical training as an internal medicine resident in New York City.

*Q: Looking back, what do you consider your main contributions to the field of public health?*

A: During my time with NIAID I wore three hats and was fortunate enough to make contributions in their corresponding areas. First, as a basic and clinical scientist where I was able to make individual contributions in inflammatory and autoimmune diseases research. With the NIH, I started out as a fellow in infectious diseases and clinical immunology and between 1972 and 1981, wearing my clinical immunologist hat, I developed highly effective therapies for the vasculitis syndromes such as granulomatosis with polyangiitis and established something of a reputation in that area. I also worked on the immunopathogenic mechanisms of HIV infection which supported the development of HIV treatments.

*Q: When did you start working on HIV?*

A: When we started to see the first few cases in the United States. That was in the summer of 1981. I became fascinated with the disease and decided to devote myself to understanding and responding to it. It really was a turning point for me, both in terms of my career and my life.

“[We need] public health leaders and public health communicators grounded in science.”

*Q: Can you say more about that?*

A: Well, to begin with you have to remember we knew next to nothing about the disease other than the fact that it appeared, initially at least, to affect only young, otherwise healthy men who have sex with men. We were convinced that we were dealing with a viral infection, knew that it was transmissible and was destroying people’s immune systems, but we did not know anything about the virus itself. I was fascinated by the different questions raised, but also deeply affected by the plight of the young men who were getting sick and dying. Many of my friends and mentors advised me against pursuing a disease that some thought might actually disappear after a while, especially given that I had already established a reputation as a young researcher in other areas. Of course, HIV did not go away, becoming a huge, global public health threat. Engaging with it took me beyond the walls of the lab and the hospital ward into the broader public health space which was all new and very interesting to me. Then, in 1984 the job of NIAID director opened up – and I guess this gets us to my second hat – and several colleagues and mentors encouraged me to apply for the position. I was reluctant to take it on because it involved administrative aspects, but I am glad I did because I ended up achieving some things that would not have been possible for me otherwise.

*Q: Can you talk about some of them?*

A: For example, driving resources into infectious disease research. When I took over in 1984, NIAID was a small, relatively secondary institution at the NIH, with a budget of about $350 million United States (US) dollars. Over the 38 years of my tenure, I built it up into an international powerhouse of infectious disease research with a budget of US$6.3 billion. During that time, I also set up the Division of AIDS to develop and implement the national research agenda to address the HIV/AIDS epidemic. The division became a major component of the institute and, in collaboration with industry, played a key role in developing the drugs that have transformed the lives of people living with HIV worldwide.

*Q: What about your third hat?*

A: I started wearing that one in 1984, the year I took over as NIAID director. President Reagan needed a briefing on HIV and the White House reached out to me as one of the most visible experts working on it. So, I went in and gave the briefing and Reagan, as good a man as he was, was not as responsive as I would have hoped. However, his Vice-President, George H W Bush, was. In fact, the Vice-President took a keen interest in the disease and what I was doing at NIAID. What’s more we really got along, later becoming good friends. I told him that the disease was going to be a global pandemic and that we needed to step up our response and he said that if he became president, he would give it the attention it deserved and would call on me for advice. And sure enough, he did become president in 1993 and I convinced him to dramatically increase the resources that the United States put into HIV. That started a resource mobilization effort that can be said to have culminated in President George W Bush’s PEPFAR (President's Emergency Plan for AIDS Relief) initiative in 2003, a plan I helped design. To date, PEPFAR has invested close to US$100 billion in the global HIV response, saving some 20 million lives, and preventing millions of HIV infections in more than 50 countries.

*Q: You went on to advise a total of seven United States presidents, from President Reagan to President Biden. How would you characterize that advisory role?*

A: In simple terms I would say it was delivering unvarnished truths about the public health issues with which different presidents were concerned, ranging from HIV to initiatives to bolster medical and public health preparedness against emerging infectious disease threats such as pandemic influenza and COVID-19. And even though it might not always be something they wanted to hear they knew they were getting the straight scoop from me and they valued that. You have to understand that adviser to the president was not an established NIAID function; it was something I started doing with President Reagan and there was a kind of domino effect with the other presidents reaching out for advice and guidance.

*Q: Were all the presidents equally receptive to the truths you delivered?*

A: With the exception of President Trump, I would say yes. It was different with him, and that difference crystallized around the COVID-19 response. For the first couple of months, he listened to me, and we had some constructive discussions about what our response should be, but then, unfortunately, he started taking advice from people who were saying things that were demonstrably untrue. For example, that drugs such as hydroxychloroquine were effective treatments for the disease. He was also saying that the virus would disappear like magic. I had to tell him that these things were not true. And not just him, I told the whole country, often openly contradicting what he had said.

“[The] evidence is the evidence. If we lose sight of that, we are all in big trouble.”

*Q: How hard was that?*

A: It was difficult and there were clearly easier options. I could have stayed in my role and kept quiet, for example, or simply left. But the first option would have made me complicit, and the second would have just meant that the President would bring in some “yes person” to agree with everything he said. In the end I felt that, because of my responsibility to myself as a scientist and to the American public, I had to be honest and open.

*Q: Were you ever afraid of what that would expose you to?*

A: I was certainly concerned, and not just for myself but for my family. Standing up to the President made me public enemy number one as far as the far-right political elements were concerned. And even to this day I am being attacked viciously by those same elements, which remain completely loyal to him. But I really did not feel I had a choice. The truth is the truth, and the scientific evidence is the evidence. If we lose sight of that, we are all in big trouble.

*Q: How hard was it to get across helpful public health messaging about COVID-19 in the face of rapidly evolving evidence regarding the transmissibility of SARS-CoV-2 or, for example, the relative health harms of exposure to the pathogen versus shutting down the economy?*

A: It was challenging, especially in the first several months, and even beyond. It is very difficult to get across the idea that messages you shared in January might have to change in March because of new information. Unfortunately, many people interpret that as flip-flopping and lose confidence in the science underpinning the messages, while the politically minded use it to advance whatever their agenda happens to be. The truth is that changing positions or the hypotheses on which they are based is an inherent part of the scientific method. Science is a self-correcting discipline, and this self-correction is one of its greatest strengths. So, yes, it was very difficult to communicate around this issue. I did my best but clearly was not completely successful. Going forward, we scientists, especially in the field of public health where so much of what is said is public facing and open to debate regarding different trade-offs, will have to do better at that.

*Q: You mentioned pursuing interests outside the federal government. Will any of these concerns feed into those?*

A: They almost certainly will, since my plan is to write and teach and lecture, drawing on the extraordinary experiences I have had both as the director of the largest infectious disease research institution in the world, and as an adviser to seven US presidents. My goal is to try to inspire a younger generation of scientists and would-be public officials to consider a career in the public health space. We face enormous public health challenges, including the emergence of new pathogens and the increasing antimicrobial resistance of pathogens, and enormous opportunities in related fields, including those inherent in the rapidly advancing biotechnologies and the harnessing of artificial intelligence. There has never been a greater need for public health leaders and public health communicators grounded in science.

